# Longitudinal variability of time-location/activity patterns of population at different ages: a longitudinal study in California

**DOI:** 10.1186/1476-069X-10-80

**Published:** 2011-09-20

**Authors:** Xiangmei Wu, Deborah H Bennett, Kiyoung Lee, Diana L Cassady, Beate Ritz, Irva Hertz-Picciotto

**Affiliations:** 1 Department of Public Health Sciences, University of California, Davis, CA, USA; 2 Graduate School of Public Health and Institute of Health and Environment, Seoul National University, Seoul, South Korea; 3 Department of Public Health Sciences, University of California, Los Angeles, CA, USA

**Keywords:** time-activity, web survey, longitudinal variation, intra- and inter-individual variation, exposure assessment

## Abstract

**Background:**

Longitudinal time-activity data are important for exposure modeling, since the extent to which short-term time-activity data represent long-term activity patterns is not well understood. This study was designed to evaluate longitudinal variations in human time-activity patterns.

**Method:**

We report on 24-hour recall diaries and questionnaires collected via the internet from 151 parents of young children (mostly under age 55), and from 55 older adults of ages 55 and older, for both a weekday and a weekend day every three months over an 18-month period. Parents also provided data for their children. The self-administrated diary and questionnaire distinguished ~30 frequently visited microenvironments and ~20 activities which we selected to represent opportunities for exposure to toxic environmental compounds. Due to the non-normal distribution of time-location/activity data, we employed generalized linear mixed-distribution mixed-effect models to examine intra- and inter-individual variations. Here we describe variation in the likelihood of and time spent engaging in an activity or being in a microenvironment by age group, day-type (weekday/weekend), season (warm/cool), sex, employment status, and over the follow-up period.

**Results:**

As expected, day-type and season influence time spent in many location and activity categories. Longitudinal changes were also observed, e.g., young children slept less with increasing follow-up, transit time increased, and time spent on working and shopping decreased during the study, possibly related to human physiological changes with age and changes in macro-economic factors such as gas prices and the economic recession.

**Conclusions:**

This study provides valuable new information about time-activity assessed longitudinally in three major age groups and greatly expands our knowledge about intra- and inter-individual variations in time-location/activity patterns. Longitudinal variations beyond weekly and seasonal patterns should be taken into account in simulating long-term time-activity patterns in exposure modeling.

## Background

Population time-activity data are an essential component for modeling exposures to contaminants in the environment. Since many health outcomes are influenced by chronic exposures, exposure assessment simulations ideally should cover extended periods (e.g. months or years) [1,2]. However, most previous studies have collected only short-term data [3-6]. Seventy-five percent of the data in the Consolidated Human Activity Database (CHAD), which houses time-activity data from a large number of studies, are single-day diaries collected in cross-sectional studies [7]. The extent to which short-term time-activity data represent long-term activity patterns is not well understood.

The scarcity of longitudinal time-activity data is in part due to the high cost of data collection. Only a limited number of studies have collected time-activity data on multiple days from individual participants in order to evaluate the intra- and inter-individual variations of time-location/activity patterns [2,7-13]. Intra-individual variation refers to the variability of time-location/activity patterns of a person's activities, e.g., by time-of-the-day, day-of-the-week, season, and weather. Inter-individual variation refers to variability between people and often can be attributed to demographic and socio-economic factors, i.e., sex, age, race/ethnicity, education, income, etc. [12,14]. Previous studies mostly focused on short-term temporal variability by time-of-the-day, by day-of-the-week and/or across seasons. Furthermore, given the high cost of collecting longitudinal data, studies usually targeted small cohorts restricted to a certain age group. Recent studies have shown that multi-day diaries lead to more accurate estimates of time allocation with no appreciable deterioration of data quality [15]. Therefore, there is a pressing need for longer-term data from population-based cohorts, allowing us to evaluate longitudinal variability of time-location/activity patterns.

As a part of the Study of Use of Products and Exposure Related Behavior (SUPERB), we collected time-location-activity diaries and questionnaires for children, parents of young children, and older age California residents over a one and a half year period using internet-based surveys to examine the intra- and inter-individual variability and longitudinal variation of their time-activity patterns [16]. We also evaluated the impact of demographic and socioeconomic factors such as age, sex and employment status. This study provides critical information on long-term human activity patterns for modeling of exposure and risk assessment.

## Methods

Details of our study design and a detailed overview of the full study data collection methods have been reported in Hertz-Picciotto et al. (2010) and Wu et al. (2011) [16,17]. Below we provide a brief description of our approach for the longitudinal web-based surveys.

### Study population

SUPERB enrolled 655 households in a telephone survey to assess the resident's time use, eating habits, and consumer products usage [16]. From the telephone survey cohort, a subset of 250 households was chosen to complete self-administered web surveys that covered the same subject matter as the telephone interview. Only subjects able to complete the web survey in English were recruited. Data were collected from two sub-cohorts. The first included 186 households in northern California having at least one child < 8 years of age, and we enrolled one parent and one child (not necessarily the youngest one) from each household. These households were selected from birth certificate records of children born between 2000 and 2005. The second cohort included 64 households of older adults (mostly ≥55-year-old) living in the southern part of California's Central Valley. These households were randomly selected from housing units listed in County tax assessor records.

### Data collection

Participants completed multiple internet surveys on a monthly basis over an 18- or 15- month period to capture the longitudinal and seasonal variations of time-activity and other exposure-related behavioral patterns. Specifically, if during one month they provided a consumer product use survey, they would be asked to complete a weekday food and time-activity recall the next month, and to provide a weekend-day food and time-activity recall the third month; then this cycle of data collection started over again in the fourth month. The time-activity portion included a diary collecting 24-hour recall of yesterday's activities and a structured questionnaire. A small number of participants (N = 20) joined the study too late to complete the full 18-months of surveys and completed only 15-months of surveys. We will refer to each three month cycle as a wave; thus, participants were asked to report for 5 or 6 waves. Parents responded for their children due to concerns about the amount of reading required in the web surveys.

We provided the equipment, services, and an in-person orientation to the computer and the web survey for 12 participants who lacked a computer or internet service. Participants accessed the survey through a study website with a unique ID and password. They received periodic e-mail reminders about upcoming survey elements, and a thank you e-mail was sent upon completion of each survey. Study participants received an incentive for their participation. In order to improve retention in the study, participants were also entered into a raffle each time they completed a web-based survey.

The 24-hour time-activity diary focused primarily on determining the amount of time spent in different types of locations with much less information asked about activities, in order to minimize participant burden. The location categories were primarily compiled from the National Human Activity Pattern Survey (NHAPS) study and the California Study of Children's Activity Patterns survey study [4,5]. Approximately 30 locations were included, as listed in Table 1. Various modes of transportation were also included. The limited information on activity we obtained allowed us to differentiate between sleeping, working (paid/unpaid), play (vigorous or not, for children), and awake doing other things. The questionnaire collected additional information on particular activities that could potentially lead to higher levels of exposure as this was less burdensome than including such activities into the diary (e.g., moderate/vigorous activity, cooking a meal on a stove), as well as activities that occurred less frequently (e.g. pumping gas, barbequing, going to a night club). We also specifically asked about frequency of doing moderate and vigorous activities outside for at least 30 minutes, as these activities are relevant for exposure to ambient air pollution.

**Table 1:** Time spent in Microenvironments (minute/day) (doers only)

Microenvironment	Children (N = 949 person-day)					Parents of young children (N = 941 person-day)					Older adults (N = 480 person-day)				
	
	%doers	**Mean**^**a**^	SD	Med	**90**^**th**^**%**	%doers	**Mean**^**a**^	SD	Med	90^th^%	%doers	**Mean**^**a**^	SD	Med	90^th^%
Residential	99.7%	1182	217	1215	1440	99.7%	1139	235	1188	1420	99.6%	1163	243	1220	1440

Own Home	99.2%	1134	243	1165	1440	99.3%	1101	254	1140	1415	97.9%	1134	260	1195	1440

School/Childcare	32.3%	325	164	345	535	20.2%	154	185	80	430	5.4%	194	148	165	480

Transit	81.3%	75	79	55	140	86.5%	89	77	75	160	82.9%	99	95	70	195

Places for Work, Shopping, Eating, Errands	34.2%	91	77	75	175	59.1%	215	198	120	530	56.0%	171	188	95	530

Office building	2.6%	136	180	50	510	19.0%	359	201	450	555	15.6%	303	258	255	575

Food Store	8.7%	45	32	40	75	15.4%	46	43	40	75	16.5%	45	44	30	80

Multipurpose Store	5.4%	69	40	60	120	8.9%	71	64	53	135	14.8%	56	39	45	120

Other Store/Shopping Mall	8.0%	91	61	80	185	11.8%	98	74	85	210	10.8%	69	51	60	130

Restaurant	14.1%	69	38	60	110	19.7%	85	87	60	140	22.7%	75	45	70	120

Beauty Salon	0.7%	45	26	35	85	1.4%	66	47	60	130	0.4%	25	7	25	30

Medical Facility	3.2%	77	91	60	120	6.1%	192	224	75	545	3.5%	175	225	60	500

Industrial Facility	0.3%	35	23	30	60	0.4%	323	268	345	590	1.3%	91	93	50	270

Auto Related Repair Shop	0.8%	25	33	10	105	1.7%	167	235	25	600	1.7%	48	34	45	110

Dry Cleaners	0.0%	.	.	.	.	0.2%	10	0	10	10	0.2%	10	.	10	10

Bar or Nightclub	0.0%	.	.	.	.	0.0%	.	.	.	.	0.2%	20	.	20	20

Various Other Locations	39.6%	164	173	120	290	42.9%	162	167	110	300	44.2%	211	214	150	455

Public Park/Beach/Golf Course	19.3%	127	102	100	240	18.0%	131	115	100	240	13.8%	160	106	133	310

Other Indoor General	5.9%	203	309	108	480	8.3%	156	233	85	410	15.8%	188	267	93	480

Gym, Health Club	8.0%	84	53	63	150	10.9%	85	46	70	135	4.8%	136	188	90	180

Public Bldg/Museum/Theatre	4.1%	99	57	95	180	4.8%	95	63	75	180	6.3%	127	114	80	275

Amusement Park or Zoo	1.4%	223	123	205	410	0.9%	266	138	230	500	0.0%	.	.	.	.

Religious Institution	7.1%	149	78	120	260	7.2%	148	84	120	275	10.2%	167	104	150	285

Farm^b^	0.3%	133	121	120	260	0.5%	339	303	255	745	3.3%	167	168	95	375

Hotel or Motel	0.7%	485	247	490	760	1.2%	363	257	390	660	0.6%	630	65	660	675

Construction Site	0.0%	.	.	.	.	0.0%	.	.	.	.	0.6%	60	0	60	60

Sleep (activity)	100%	669	96	660	780	100%	542	112	540	675	100%	528	130	510	665

Work (activity)	0.0%	.	.	.	.	32.6%	397	188	450	595	26.9%	347	216	330	600

^a ^Arithmetic means are presented. SD - standard deviation. Med - median.

^b ^Older adults all came from largely rural counties, while the younger ones were predominantly from urban areas.

The web surveys were conducted between October 2007 and September 2009. Research protocols and consent forms of this study were approved by the Institutional Review Board of the University of California at Davis.

### Data Analysis

A full evaluation of the web-survey method and data cleaning have been described previously in Wu et al. [17]. Reliability was evaluated by examining the consistency of the number of records and number of location changes reported in a diary, and the validity was tested by comparing with data collected through telephone interviews of the same respondent. Both the reliability and validity were acceptable for this web survey method. The performance of the web surveys collected was evaluated in terms of, for example, the survey completion rate and the percent of surveys with an unreasonable amount of time reported for sleeping. We observed compliance issues in a small number (< 3%) of web diaries, and these diaries were not included in the analysis. Diaries were considered sufficiently complete for inclusion: if (a) they contained three or more time-location-activity records; and (b) information was missing for less than 3-hours per day.

**Figure 1:**
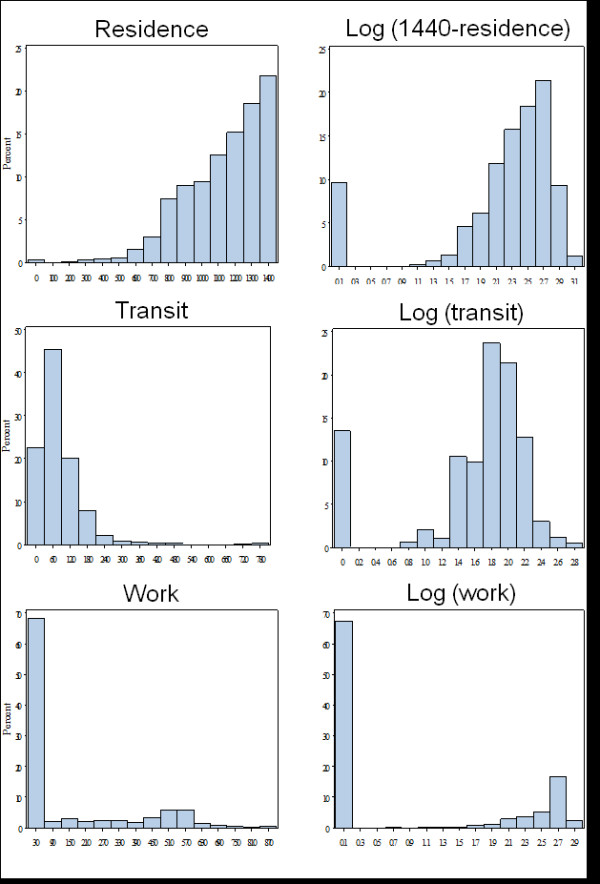
**Examples of the distribution and transformation of time-location/activity data (Time spent at a residence, in transit and at work in original and log-transformed distribution)**.

For the diary data, we summarized the time spent in different microenvironments and in activities for each age group. The time spent in most of the microenvironments/activities can best be represented as a mixed distribution, with a large number of diaries having no time in a particular microenvironment and the rest of the data following a normal or log-normal distribution (Figure 1). A mixed-distribution mixed-effects model was employed to evaluate the intra- and inter-individual variability of time spent in certain microenvironments/activities. The details of this statistical method can be found in Tooze et al. (2002) and Xie et al. (2004) [18,19]. Briefly, an occurrence variable (O) that was modeled by logistic regression specifies whether or not people spent time at a location or in an activity; and for the non-zero values, a duration variable (D) that was modeled by normal or lognormal regression specifies how long people spent at a location or on an activity. Time spent in any residence fell into a different distribution, with a portion of diaries reporting 24 hours spent at any residence and the rest following a log-normal distribution. Therefore, time spent at a residence was subtracted from 24, such that values of zero represent people staying at any residence all day and the remainder being represented by a log-normal distribution for time spent away from any residential environment. The same procedure was used for time spent at one's own home. To examine activity patterns of individuals, we included day-type (weekday vs. weekend), season (May to October defined as warm season vs. November to April as cool season, according to the climate in California), the time variable (1^st^-6^th ^wave), sex, and age as covariates in the model. Random effects of the two parts of the model were assumed to be correlated. Intraclass correlation coefficients (ICC), the ratio of between-subject variance to the sum of between- and within-subject variance, were calculated as an indicator for the consistency of an individual's activities. A SAS Macro, MIXCORR, developed by Tooze et al. (2002), was used for this analysis [18]. For general microenvironments in which people spent time every day, e.g., home, we broke down the population by age group. For specific microenvironments for work, shopping, eating and errands and various other locations, participants from different age groups were pooled and analyzed together because of the small number of events of this kind. Only participants who completed diaries in more than one wave were included in these analyses.

For information collected by questionnaires, duration and frequency of exposure-related activities were also summarized by age group. As with the diary data, we characterized variation by day-type, season, and wave. Some demographic factors, such as sex, employment status (for adults), and age (for children), were also examined. Duration and frequency variables both had continuous distributions of non-zero values, presenting a normal or log-normal distribution. In such cases a generalized linear mixed-effects model was used. Only the questions about the use of a computer, watching TV, and barbequing allowed for a zero answer. Given that a significant fraction of diaries had a zero value for these three activities, a mixed-distribution mixed-effects model was used to analyze these data. Only participants who completed the questionnaire in more than one wave were included in this analysis. All analyses were conducted using SAS 9.2. Statistical significance was set as α = 0.05 (two-sided).

## Results

A total of 206 households provided any time-activity data, which encompassed 150 young children (mostly under 8 years of age), 151 parents of young children (mostly under 55 years of age), and 55 older adults (mostly above 55 years of age) (see Additional File 1). Overall, we collected 1,953 diaries (with separate diaries for parents and children) and 985 questionnaires for parents of young children (parents' questionnaires covered children's activities). We also collected 504 diaries and 509 questionnaires from older adults. Of note is that the SUPERB study was designed to put an emphasis on collecting data from households with young children and older adults, thus, the study cohort included a high percentage of stay-at-home parents (38% of the parents) and retired people (49% of older adults), who were not employed. Among the 55 older adults, 63% rated their overall health condition as "Good" to "Excellent".

### Assessment of 24-hour time-activity diaries

We present time spent in each of five major microenvironments and for subcategories of selected locations and activities for each age group in Table 1. Participants spent approximately 80% of their time (19.2 hours on average) at their own home, and about half of the time at home was reported as sleeping; children slept longer than adults. On average, participants spent 1-1.5 hours per 24-hour recall in transit. A smaller portion of time was spent at places for work, shopping, eating, and errands, with medians ranging from 1.25-2 hours, considering both weekday and weekend days. Using the terminology of the NHAPS, we will refer to people who visited a location or conducted an activity as "doers". We found that the percentage of doers ranged from < 1% for dry cleaning establishments to 18% for restaurants. Adults spent more time than children at places for working, shopping, eating and errands. Older adults generally spent similar or less time than parents of young children in such places.

Several factors appeared to influence the time spent in selected microenvironments and activities (Table 2). For each location/activity and age group, results for both occurrence and duration parts of the mixed-distribution mixed-effects models are presented. The only exception is the time spent sleeping, since sleep time followed a normal (for children) or lognormal (for adults) distribution. Variation by each factor found to influence how time was spent is summarized below.

**Table 2:** Variation of time spent in selected microenvironments or activities (Only results with statistical significance *p *< 0.05 are shown.)^a^

Variables	Age group	**Model**^**b**^	**Daytype**^**c**^	Season	Longitudinal	**Sex**^**d**^	**Age**^**e**^	ICC	O/D correlation
	Children	O	WE > WD	cool > warm				0.24	r = 0.82 *p *= 0.0003
		
		D	WE > WD				decreasing	0.15	
	
Home^f^	Parents	O					decreasing	0.20	r = 0.88 *p *< 0.0001
		
		D	WE > WD					0.25	
	
	Older	O						0.39	r = 0.50 *p *= 0.0734
		
		D	WE > WD					0.17	

	Children	O	WD > WE	warm > cool				0.20	r = 0.48 *p *= 0.0285
		
		D			increasing			0.16	
	
Transit	Parents	O		warm > cool	increasing		increasing	0.25	r = 0.72 *p *= 0.0013
		
		D	WD > WE					0.15	
	
	Older	O						0.27	r = 0.49 *p *= 0.1289
		
		D		warm > cool				0.14	

Places for work, shopping, eating, errands	Children	O	WE > WD					0.10	r = -0.12 *p *= 0.6544
		
		D						0.21	
	
	Parents	O	WD > WE					0.24	r = 0.43 *p *= 0.0187
		
		D	WD > WE			M > F		0.30	
	
	Older	O	WD > WE					0.14	r = 0.50 *p *= 0.0983
		
		D	WD > WE				decreasing	0.19	

Other locations	Children	O	WE > WD	warm > cool				0.11	r = 0.45 *p *= 0.2407
		
		D	WE > WD					0.07^g^	
	
	Parents	O	WE > WD	warm > cool			increasing	0.19	r = 0.49 *p *= 0.0990
		
		D	WE > WD	warm > cool				0.09^hg^	
	
	Older	O						0.39	r = 0.69 *p *= 0.6360
		
		D	WE > WD					0.01^g^	

Sleep (activity)	Children		WE > WD		decreasing		decreasing	0.24	N/A
	
	Parents		WE > WD				decreasing	0.18	N/A
	
	Older		WE > WD					0.21	N/A

School	C	O	WD > WE				increasing	0.20	r = 0.47 *p *= 0.0738
		
		D	WD > WE			F > M		0.38	

Work (activity)	All adults	O	WD > WE		decreasing			0.41	r = 0.61 *p *= 0.0021
		
		D	WD > WE				decreasing	0.43	

Office	All	O	WD > WE					0.41	r = 0.97 *p *< 0.0001
		
		D	WD > WE	cool > warm				0.74	

Food store	All	O				F > M	increasing	0.14	r = -0.72 *p *= 0.0082
		
		D	WE > WD					0.18	

Multipurpose store	All	O			decreasing			0.20	r = 0.16 *p *= 0.6001
		
		D			decreasing			0.20	

Other store/Shopping mall	All	O	WE > WD				increasing	0.17	r = -0.44 *p *= 0.1130
		
		D			decreasing			0.32	

Restaurant	All	O	WE > WD				increasing	0.24	r = 0.43 *p *= 0.0286
		
		D	WE > WD					0.22	

Public park	All	O	WE > WD	warm > cool			decreasing	0.28	r = -0.19 *p *= 0.5821
		
		D	WE > WD	warm > cool		M > F	increasing	0.06^g^	

Health club	All	O	WD > WE	cool > warm				0.43	r = -0.68 *p *= 0.1107
		
		D	WE > WD			M > F	increasing	0.13^g^	

^a ^Only participants who completed diaries in two or more waves were included in this analysis.

^b ^Given that the time-location/activity data clump at zero, mixed-distribution mixed-effects model was used except for time spent on sleep. The mixed-distribution mixed-effects model consists of two parts: whether an individual spent time at a location (occurrence - O) and how long he/she spent at that location (duration - D) (Tooze et al., 2002; Xie et al., 2004). The generalized linear mixed-effects model was used for time spent on sleep.

^c ^weekday (WD) vs. weekend (WE). ^d ^male (M) vs. female (F). ^e ^increasing: older > younger; decreasing: older < younger

^f ^Time spent at respondents' own home was transformed using (1440 - time spent at home), thus a zero value means an individual spent a whole day at home. ^g ^Random effect is not statistically significant at α = 0.05.

#### Day-Type variation

Day-type (weekday vs. weekend day) appeared to be the primary factor influencing time-location/activity patterns. As expected, for time spent in school, office, and at work (activity), both the O and D parts of the models are positive and statistically significant, suggesting that people were more likely to be in these places on a weekday than on a weekend, and that they spent more time in schools, offices, and at work on weekdays than on weekend days. In contrast, the coefficients of the O and/or D models are negative and statistically significant for time spent in food stores, shopping malls, restaurants, and public parks, meaning that people were more likely to go to these places and/or spent a longer time there on a weekend day than on a weekday. Similarly, children and their parents were more likely to spend a whole day at home on a weekend than on a weekday, and the time they spent at home was longer on weekend than on a weekday (Table 2).

#### Seasonal variation

Activities occurring outside the home showed greater seasonal variation. People were either more likely to travel (young children and their parents) or spent more time in transit (older adults) in the warm than in the cool season. The same trend was observed for frequenting public parks. Young children were more likely to spend whole days at home in the cool than in the warm season. We also found that people spent more time in the office in the cool season and were more likely to go to a health club during the cool season.

#### Longitudinal variation over study period

For most of the locations/activities, we observed no trends over time during the study period (Table 2). We did find that young children slept less over time, perhaps because they eliminated a nap or decreased nighttime sleep needs as they aged. We also observed a trend for increasing likelihood of transit for parents of young children and decreasing occurrence and/or duration of time spent working and shopping during the study. Interestingly, these changes were consistent with gas price fluctuation (a dramatic price drop in the fall of 2008) and the impact of the economic recession (from early 2008 extending beyond the study's end in September 2009).

#### Variation by demographic factors

In general, fathers of young children spent more time at places for work, shopping, eating and errands than mothers; however, when all adults were considered, females were more likely to visit a food store than males. Male doers spent more time in other locations, like public parks and health clubs. In addition, girls who went to school or daycare spent more time at school than boys.

Age effect was also examined within each age group. Older parents of young children were less likely to spend the whole day at home and more likely to spend time in transit and other locations, like parks and museums, than younger parents of young children. Time sleeping decreased with age for both young children and parents of young children. Among the older adult doers, time spent at work, shopping, eating and running errands decreased with age. Older adults generally spent less time working than adults who were younger and had children.

#### Inter-individual variation

Inter-individual variation was measured by the random effect, more specifically, the intraclass correlation coefficients (ICCs). The random effect was significant for most locations/activities, suggesting considerable difference in time-location/activity patterns between individuals. The ICCs for most of the location/activity categories ranged between 0.10 and 0.43. Higher ICCs were observed for time periods that children spent in school and adults spent in the office and working, corresponding to the routine nature of work and school, which translates into less variability within an individual. Instead, time spent at school or work varied across individuals.

#### Correlation between occurrence of events and duration of events

The random effects of the O and D parts of the mixed-distribution mixed-effects model were assumed to be correlated, i.e., allowing potential correlations between the likelihood and duration of an individual's activity. These correlations were positive for time spent in transit, in the office and school, at work, and in restaurants (Table 2). In other words, microenvironments that were visited more often were ones where people tended to spend longer time, and vice versa. Negative correlation was observed for time spent in food stores, suggesting that the more often people went to food stores, the shorter the time they spent during each visit.

### Assessment of internet-based questionnaires

Separate from the 24-hour recall diary, questionnaires asked about selected exposure-related activities. Participation in and duration of these activities, e.g., moderate or vigorous outdoor activity, being in the garage, and using stove, were obtained for each recall day. Duration of some activities that were recalled is shown by weekday and weekend day for each age group in Table 3.

**Table 3:** Duration and frequency of exposure-related activities (doers only)

Activities	Children (N of questionnaires = 985)					Parents of young children (N of questionnaires = 985)					Older adults (N of questionnaires = 509)				
	
	% doers	Mean	SD	Med	90^th^	% doers	Mean	SD	Med	90^th^%	% doers	Mean	SD	Med	90^th^%
**Duration of daily activity (minute/day)**															

**Weekday**	N = 511					N = 511					N = 258				

Any moderate activity	91%	192	128	180	360	92%	219	173	180	480	89%	168	128	125	330

Moderate outdoor activity > 30 minutes	56%	77	77	60	135	33%	85	79	60	180	31%	118	123	60	270

Any vigorous activity	77%	126	83	120	240	28%	79	53	60	140	28%	114	105	70	210

Vigorous outdoor activity > 30 minutes	56%	87	88	60	150	15%	104	136	60	180	14%	97	63	80	190

Being in the garage	19%	17	43	5	25	26%	29	98	10	30	51%	58	132	20	90

Using stove^a^	32%	36	107	15	60	65%	57	53	45	120	53%	56	67	45	120

Using computer	33%	63	76	45	120	90%	217	183	150	480	87%	160	109	125	360

Watching TV	88%	110	74	90	200	78%	110	73	120	180	93%	205	148	180	360

Being in a room with windows open	--	--	--	--	--	62%	380	280	300	780	64%	437	271	420	780

**Weekend**	N = 474					N = 474					N = 251				

Any moderate activity	92%	213	135	180	370	93%	219	160	180	480	88%	185	137	150	360

Moderate outdoor activity > 30 minutes	59%	92	81	60	180	42%	107	90	75	190	34%	101	88	60	210

Any vigorous activity	74%	142	98	120	300	22%	106	101	60	240	25%	135	107	120	255

Vigorous outdoor activity > 30 minutes	50%	110	75	90	210	14%	119	121	73	225	15%	115	63	105	240

Being in the garage	23%	25	81	10	30	31%	24	67	10	60	48%	46	104	20	75

Using stove^a^	29%	32	95	15	60	58%	62	50	60	120	52%	69	99	45	135

Using computer	38%	79	110	60	140	74%	111	119	60	240	79%	114	79	90	225

Watching TV	87%	121	84	120	240	81%	129	95	120	240	89%	238	168	188	480

Being in a room with windows open	--	--	--	--	--	71%	401	268	325	780	66%	448	273	390	780

**Frequency of weekly activity (time/week)**															

Moderate outdoor activity (> 30 minutes)*	84%	6	4	5	10	65%	4	4	3	7	49%	5	4	4	8

Vigorous outdoor activity (> 30 minutes)*	81%	6	4	5	10	51%	4	4	3	6	39%	3	3	3	7

Purchasing gas at a gas station	--	--	--	--	--	93%	1	1	1	2	88%	1	1	1	2

Food store visit	85%	1	1	1	3	96%	2	1	2	3	95%	2	2	2	3

**Frequency of monthly activity (time/month)**															

Multi-purpose store visit	92%	2	2	2	4	97%	3	2	2	5	95%	3	2	3	5

Other store visit	83%	2	3	1	4	89%	3	4	2	5	82%	4	8	2	8

Barbeque	29%	2	2	2	5	44%	3	4	2	6	44%	4	4	3	12

Visiting bar/nightclub	--	--	--	--	--	16%	1	1	1	2	11%	1	1	1	2

^a ^For children, approaching stove when adults were cooking.

* An outlier in parent of young children with the frequency of 100 times/week was excluded.

Participants, both adults and children, engaged in some sort of moderate activity on the majority (~90%) of recall days, and children were more likely to engage in vigorous activity or aerobic exercise than adults (77% vs. 28% of all recall days). Moderate activities were defined in the questionnaire by providing examples of walking, cleaning, food preparation, and other activities generally requiring standing up. Examples for vigorous activities in the questionnaire were running, bicycling, digging, and building. The "moderate" and "vigorous" activities were chosen such that they would be easily understood by respondents, but do not necessarily correspond to strict definitions based on available metabolic equivalent (MET) intensity levels for physical activities (Ainsworth et al., 2000). Thus compared to stricter definitions of moderate and vigorous physical activities, we may over-estimate the likelihood and duration of such behaviors.

Adults used computers on 87-90% of all weekdays and 74-79% of all weekend days, while young children used the computer less often, only on ~30% of all recall days. Older adults generally spent more time watching TV than parents of young children. Eighty-seven percent of parents reported their children washed their hands during the day, and on average, children washed their hands 4 to 5 times per day. Frequency of less common activities (e.g., those reported to happen weekly or monthly) are also presented. The majority (95%) of adult respondents went to food and multipurpose stores routinely, on average, 2 times per week to food stores and 3 times per month to multipurpose stores. Only a small number (14%) of adults (including both parents of young children and older adults) went to a bar or nightclub, with answers ranging from 1 to 4 times in a typical month. Variation of time spent on selected activities is summarized in Table 4 and discussed below.

**Table 4:** Variation of activities collected by questionnaires^a ^(Only results with statistical significance *p *< 0.05 were shown.)

	Age group	**Model**^**b**^	**Day-type**^**c**^	**Season**^**d**^	Longi- tudinal	**Sex**^**e**^	Employment **Status**^**f**^	Age of **Children**^**g**^	ICC
**Daily activity: time spent on the activity per day (minute/day)**									

Any moderate activity	Children		WE > WD					O > Y	0.36

	Parents			warm > cool			UE > E		0.34

	Older								0.26

Moderate outdoor activity (> 30 minutes)	Children		WE > WD	warm > cool	decreasing				0.14

	Parents		WE > WD						0.15

	Older								0.19

Any vigorous activity	Children			warm > cool	decreasing	M > F			0.35

	Parents						UE > E*		0.32

	Older								0.25

Vigorous outdoor activity (> 30 minutes)	Children		WE > WD	warm > cool	decreasing				0.09

	Parents					F > M*			0.16

	Older								0.16

Being in the garage	Children		WE > WD						0.21

	Parents								0.45

	Older								0.28

Using stove (adults) or approaching stove while someone cooking (children)	Children								0.44

	Parents								0.40

	Older								0.28

Using computer^b^	Children	O			increasing			O > Y	0.45

		D	WE > WD		increasing	M > F			0.37

	Parents	O	WD > WE						0.41

		D	WD > WE			M > F	E > UE		0.31

	Older	O	WD > WE						0.43

		D	WD > WE		decreasing		E > UE		0.31

Watching TV^b^	Children	O			decreasing			Y > O	0.52

		D	WE > WD						0.46

	Parents^p^	O				M > F			0.44

		D	WE > WD	cool > warm					0.31

	Older^p^	O							0.58

		D							0.58

**Weekly or Monthly activity: frequency (time/week or time/month)**									

Moderate outdoor activity (> 30 minutes) per week	Children		--	warm > cool					0.38

	Parents		--	warm > cool	decreasing				0.31

	Older		--			M > F			0.40

Vigorous outdoor activity (> 30 minutes) per week	Children		--		decreasing				0.40

	Parents		--						0.30

	Older		--						0.43

Purchasing gas at a gas station per week (adults only)	Parents		--						0.53

	Older		--			M > F	E > UE		0.58

Food store visit per week	Children		--	warm > cool	decreasing				0.64

	Parents		--		decreasing		UE > E		0.70

	Older		--						0.73

Multi-purpose store visit per month	Children		--		decreasing				0.57

	Parents		--		decreasing				0.64

	Older		--						0.52

Other store visit per month	Children		--						0.61

	Parents		--		decreasing				0.60

	Older		--			M > F			0.61

Barbeque per month^b^	Children^p^	O	--	warm > cool					0.61

		D	--	warm > cool					0.52

	Parents^p^	O	--	warm > cool	decreasing				0.57

		D	--	warm > cool					0.57

	Older^p^	O	--	warm > cool		M > F			0.60

		D	--	warm > cool		M > F			0.70


^a ^Only participants who completed diaries in two or more waves were included in this analysis.

^b ^The generalized linear mixed-effects model was used for most activity data with normal or lognormal distribution. Mixed-distribution mixed-effects model was used for most of the activity data clumping at zero. The mixed-distribution mixed-effects model consists of two parts: whether an individual spent time t a location (occurrence - O) and how long he/she spent at that location (duration - D) (Tooze et al., 2002; Xie et al., 2004).

^c ^weekday (WD) vs. weekend (WE). ^d ^warm (W) vs. cool (C). ^e ^male (M) vs. female (F).

^f ^Employment status of adults (for analysis of adult data only): employed (E) vs. unemployed (UE).

^g ^Children's age (for analysis of children's data only): increasing - older than 6-years-old > at 6 or younger; decreasing - older than 6-years-old < at 6 or younger.

* marginally significant; ^p ^O/D positively correlated

#### Day-type variation

Day-type variations were investigated only for activities that usually happen on a daily basis such as using a computer, watching TV, and participating in moderate outdoor activities for adult participants. Both younger and older adults were more likely to use a computer and to use it for a longer period on weekdays than on weekend days. In addition, younger adults spent more time watching TV and conducting moderate outdoor activities on weekend days than on weekdays. Compared with weekdays, children spent more time on weekend days engaged in many of the activities we asked about, including moderate and vigorous outdoor activities, being in a garage, using a computer, and watching TV.

#### Seasonal variation

Based on the diaries, people engaged in more outdoor activities in the warm season than in the cool season. For example, in the warm season participants from all age groups reported higher frequencies of barbequing, and young children and their parents spent more time with moderate activities and moderate outdoor activities for at least 30 minutes. Young children spent more time doing vigorous activities, and the reported frequency of going to the food store was higher in the warm season. Correspondingly, parents of young children spent less time watching TV in the warm season. All of these results can be found in Table 4.

#### Longitudinal Variation over study period

For several activities we observed a decreasing trend over time, mostly for young children and their parents. Parents of young children reported less frequently participating in moderate outdoor activities, going shopping, and barbequing over time. Children also spent less time on moderate and vigorous activities and went shopping less frequently over time. This echoes the decreasing occurrence and/or duration of reported time spent shopping over the 15- to 18-month observation period for the diary data.

Another interesting longitudinal trend is that, as time progressed, children were more likely to use a computer, but less likely to watch TV. This could have been due to their maturing, i.e., the youngest children may have lacked the skills to use the computer and/or may not have been permitted to use it. However, as we did not collect this information, we cannot draw conclusions about the reasons for this trend. In contrast, older adults reported using a computer for less time per day over the length of the study.

#### Variation by demographic factors

In households with young children, mothers of young children were less likely to watch TV and use computers than fathers. However, mothers spent more time on vigorous outdoor activities than fathers. We note that our population had a large number of stay-at-home mothers. Older male adults reported a higher frequency of moderate outdoor activities, purchasing gas, barbequing, and visiting other types of stores than older females. The only sex differences in activities for young children that we observed were for boys spending more time engaged in vigorous activities and using computers than girls.

Employed adults spent more time using a computer on the recall day than unemployed adults, and employed older adults bought gas more often. We also observed that unemployed parents of young children, overwhelmingly female, spent more time on moderate and vigorous activities, and visited food stores more often than employed parents.

Consistent with the longitudinal trend, children younger than 6-years were more likely to watch TV, while children older than 6 were more likely to use a computer. Older children also spent more time on the recall day on moderate activities than younger children.

#### Inter-individual variation

The ICCs for time spent on selected activities were generally moderate, ranging from 0.14 to 0.58 for activity that happened on a daily basis. ICCs were higher (0.30-0.73) for activities that happened less often, suggesting variability in activity pattern between individuals.

#### Correlation between occurrence and duration

The likelihood and frequency of using a computer and watching TV are positively correlated, namely the more likely one engaged in an activity, the longer time one spent on the activity.

### Consistency between diaries and questionnaires

We further examined the correlation between the duration of time spent in food and multipurpose stores collected in the diaries with the frequency of visiting food and multipurpose stores reported in the web-survey questionnaires. The time spent in food stores as collected by the recall diary were negatively correlated with the frequency of food store visits though this was only marginally statistically significant (r = -0.12, *p *= 0.07). Once we limited the analysis to data collected in the first wave, the correlation became stronger (r = -0.32, *p *= 0.03). This is consistent with the results for the recall diaries, specifically that participants who reported more instances of going to the food store spent less time in the store. No such correlations were observed for going to a multipurpose or other type of stores.

## Discussion

This study was designed to evaluate longitudinal variations in time-activity patterns. Data collected in this study allow us to evaluate inter- and intra-individual as well as longitudinal variations in human time-activity patterns.

The time-location/activity data we collected are substantially in agreement with two large time-activity studies conducted in the U.S. The California Study of Children's Activity Patterns survey (April 1989 - February 1990) collected 24-hour recall from 1200 children under 12-years-of age, half of whom were age 6 or above [4]. This group reported that children under 12-years-of age spent 1,078 minutes/day (18 hours/day) at home and 109 minutes/day in school/childcare on average. Younger children (1 to 6-year-old) spent more time at home and less time in school than older children (6 to 11-year-olds), which echoes our results in SUPERB where children spent 1134 minutes/day (19 hours/day) at home and 105 minutes/day in school/childcare on average. The time children spent in transit was similar in both studies too, that is, 69 minutes/day in the California Children's Study vs. 61 minutes/day for children under 12-years-of age in our study on average.

**Figure 2:**
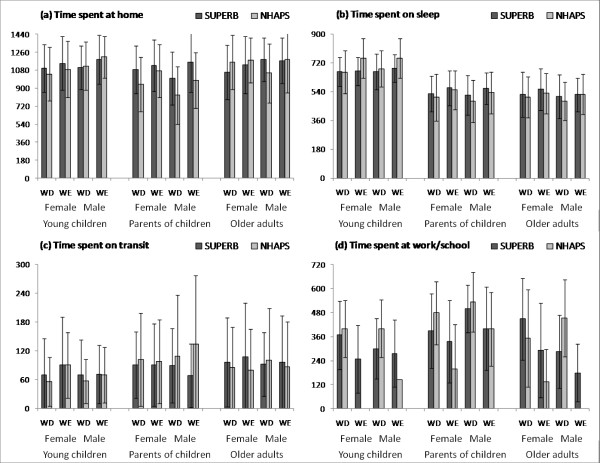
**Comparison of time-location/activity data between SUPERB and NHAPS by day-type, gender and age group**. The solid bars represent the means and the error bars represent 95% confidence intervals of the means. For NHAPS data, the sample size was 84 for children under 8-year-old, 436 for adults between 26 and 55 years old, and 251 for older adults above 55-year-old. Note that, the people in NHAPS which were compared with "parents of young children" in SUPERB were only in the comparable age group but not necessarily having young children.

A second key study is the National Human Activity Pattern Survey (NHAPS). NHAPS collected 24-hour recall from 9,368 respondents across all age groups in the U.S between October 1992 and September 1994 [5]. We compared our data with a subset of NHAPS data, those collected from respondents in comparable age groups who lived in California. Note that adults in NHAPS did not necessarily have young children. The time spent in selected microenvironments/activities reported in the two studies were generally similar, with discrepancy mainly observed for the age group of parents of young children (Figure 2). Parents of young children in SUPERB spent more time at home and less time in transit. In particular, fathers spent much less time in transit on weekends, and mothers reported shorter time spent working on weekdays but longer time on weekends compared to NHAPS adults in the same age group. Craig and Mullan (2010) also reported that parents of young children spent on average five more hours per day on child care and housework than childless men and women, and in particular, mothers spent 2.8 fewer hours working than childless women in the same age group [20]. Apart from this difference, the time allocation observed in SUPERB is similar to the range reported in NHAPS.

Our findings concerning day-type and seasonal variations are consistent with Echols et al. (1999) [12]. They examined variations in 80 individuals (primarily adults) by day-of-week (i.e., Monday through Sunday), cycle (six sampling cycles over a year), and individual. Significant day-of-week variations were observed for all seven time-location categories they examined, i.e., more time spent in transit and at work/school (both in and outdoors) on weekdays compared to more time spent at home (both in and outdoors) and other locations (both in and outdoors) on weekend days. Since they did not include season as a covariate in the model, the variations by cycle they reported were effectively the same as seasonal variability, e.g., less time spent at work/school indoors but more time spent at home outdoors and in other outdoor locations in the warm months (June to September), which are consistent with the seasonal variations of time spent at home, in transit, and in the school and office we reported for SUPERB participants.

Previous studies examining variations in time-location/activity patterns were based on very specific populations or were more restricted with regard to locations included. Our study provides additional evidence that confirms previous findings. A previous study conducted by McCurdy and Graham analyzed data on 57-60 year old males and found that day-type and season greatly influenced time spent indoors and outdoors but not time spent in motor vehicles [14]. In contrast, Frazier et al. (2009) reported little day-type and seasonal variability for time spent indoors, outdoors or in motor vehicles in a cohort of elderly aged 56-83 with chronic obstructive pulmonary disease in Los Angeles; day-type variation was stronger in a cohort of elderly (aged 65-89) in Baltimore [7]. In our study, 17% of the older participants had heart disease and 23% had asthma, while 63% of the older participants considered their overall health condition healthy. The time that older adults spent in total indoors, outdoors, or in vehicles did not significantly varied by day-type, but day-type variation was observed for time spent in specific microenvironments, i.e., home and places for work, shopping, eating or running errands. We also observed that older adults spent more time outdoors in the warm season than in the cool season (see Additional File 2).

Graham and McCurdy (2004) considered age and gender as the primary factors to define a cohort in a time-activity study [21]. We did observe statistically significant impacts of sex, age and employment status on time spent in some locations and on some activities. However, our younger adult population comprised solely of parents of young children, which may have influenced their time-activity patterns. Therefore, the variation by sex and age we observed may not be generalizable to populations of different characteristics (employment, family size).

Previous studies used ICC values, the ratio of between-subject variance to total variance, as a measure of for inter-individual variability. Xue et al. (2004) and Frazier et al. (2009), based on diaries collected daily from elementary school children and a cohort of elderly, respectively, obtained ICCs ranging from 0.10 to 0.35 for time spent indoors, outdoors and in a vehicle [2,7]. We obtained ICCs in the same range if we allocate time in a similar way (see Additional File 2). The ICCs for time spent on exposure-related activities that were asked in the web-survey questionnaire are generally higher than the ICCs for time spent in different locations that were asked in the 24-hour recall diary, indicating greater variability in activity patterns than space transition between individuals.

One of the unique contributions of the SUPERB web survey was that time-activity data was collected over an extended 18-month period, allowing us to evaluate longitudinal variation over a longer period than previous studies. We found that time-activity patterns were basically consistent over the study period, with some exceptions possibly related to human physiological changes and socio-economic factors. Results suggest that day-type is a primary source of variation for time-activity patterns, with season a second and usually predictable source; beyond these, human time-activity patterns were basically consistent over time. Therefore, for exposure modeling purposes, researchers should use cross-sectional time-activity surveys to collect baseline human activity pattern on different types of days (weekday and weekend), and then account for seasonal variations. For seasonal variations, one could either collect data in different seasons or estimate seasonal variation by incorporating known seasonal variation into the model. In addition, long-term time-activity patterns due to social and economic changes, which have not been paid attention to before, should be included. For young children and older people, physiological changes also need to be considered. We recommend use of supplemental questionnaires to collect the frequency of exposure-related activities that happen less often and therefore may not be captured on a single sampling day, e.g., vigorous outdoor activity, pumping gas, barbeque, etc.

Another contribution of our study is the investigation of intra- and inter-individual variation in many more locations and for more activities than studied before. We extended the limited number of micro-environments (home, work/school, transit and other locations) or standard activity categories (indoors, outdoors and in-vehicle). We collected longitudinal data on ~30 location/activity categories and examined the intra- and inter-individual variation in half of them, including restaurants, several types of stores, parks, health clubs, etc. Our findings thus greatly expand the current knowledge about the variation in human time-activity patterns for the three age groups.

Furthermore, we introduced mixed-distribution mixed-effects modeling to analyze time-activity data in which a large percentage of participants are assigned a zero value. Compared to the traditional non-parametric method, e.g., relying on the Kolmogorov-Smirnov test, this method provides a better solution for time-activity data that are not normally distributed, because we were able to simultaneously assess both the likelihood and the duration of the time spent in microenvironments/activities.

A limitation of this study is that participants gradually withdrew or dropped out over time, hindering the evaluation of longitudinal variation. As reported in Wu et al., out of the 206 households, 56% of parents of young children and 24% of older adults did not complete the study. A large number (84%) of parents of young children could not complete all required surveys due to limited time and family responsibility [17]. To retain maximum information, we included all valid diaries into the statistical summary, but only the participants who completed two or more surveys into the longitudinal analysis. In addition, web surveys allow participants to select any day for recall, and they may select a convenient day. Specifically, participants may select an unrepresentative day with fewer activities than typical in order to minimize reporting effort, for example, a nurse could select a weekday that he/she was not on shift for recall. We did not include the diaries with very few location/activity changes in this analysis, but this trend helps explain why participants reported longer sleep time but shorter work hours in our study, and thus may influence the estimation of intra- and inter-individual variations.

Secondly, our definitions of moderate and vigorous activities were not precise, and tend to over-estimate the activity level. According to the activity metabolic equivalent (MET) intensity levels published by Ainsworth et al. (2000), activities with METs ranging 3-6 were considered moderate and those with METs above 6 were considered vigorous. For example, walking can be light to vigorous depending on the speed (METs ranging 2.0 to 12.0), and bicycling varies from moderate to vigorous with METs of 4.0 to 16.0 [22]. Food preparation is more commonly considered to be a light activity with METs between 2.0 and 3.0. Comparisons with available data on strictly-defined moderate and vigorous physical activities (MPA & VPA) also suggest overestimation of time spent on these activities. According to the State Indicator Report on Physical Activity, 67% and 45% of adults in California are physically active and highly active, respectively [23]. Ainsworth et al. (2000) reported that adults in their 40's spent 16 min/day in MPA and 18 min/day in VPA [22]. Sallis et al. (1985) investigated moderate and vigorous physical activities among 2126 adults between 20 and 74 years old [24]. They found that 84% of respondents engaged in moderate activities and spent 50-83 minutes/day on average depending on respondents' age; 15% of respondents reported vigorous activities and spent 17-68 minutes/day on vigorous activities. Compared to their study, we over-estimated the percentages of doers of moderate and vigorous activities by around 7% and 12%, respectively, and over-estimated the time spent on moderate and vigorous activities by approximately 3 times.

Lastly, in our method evaluation step, we compared the web survey data with the time-activity data collected by telephone interview from the same respondents and obtained similar distributions of the time-allocation measured by these two methods [17]. However, since these two types of the surveys were not conducted for the same day for a respondent, such comparison does not fully establish the validity of the web survey method. More objective methods, such as Global Position System (GPS) recordings, may provide better reference values to determine the validity of the self-reported location data [25]. Gold standards for activity data are more difficult to obtain.

## Conclusions

In summary, the SUPERB web survey provides a valuable tool to assess longitudinal time-activity data in a population-based cohort and for different age groups, allowing us to examine variations in time-location/activity patterns. Consistent with previous studies, day-type and season are the major factors influencing time-activity patterns. Some longitudinal variations were also observed, possibly related to human physiological changes and socio-economic changes, which should be taken into account in simulating long-term time-activity patterns in exposure modeling.

## List of Abbreviations

CHAD: Consolidated Human Activity Database; SUPERB: Study of Use of Products and Exposure Related Behavior; NHAPS: National Human Activity Pattern Survey; O: occurrence variable; D: duration variable; ICC: intraclass correlation coefficients; MET: metabolic equivalent; MPA: moderate physical activities; VPA: vigorous physical activities.

## Competing interests

The authors declare that they have no competing interests.

## Authors' contributions

XW was responsible for analysis and interpretation of data and drafting the manuscript, working closely with DB. IHP, DC, KL, DB, and BR made substantial contributions to study design of the SUPERB and data collection, and contributed to critical review of the manuscript for intellectual content. All authors read and approved the final manuscript.

## Supplementary Material

Additional file 1 **Demographic characteristics of the participants**. The table presents the demographic characteristics of the participants in the three age groups. Click here for file

Additional file 2 **Variation of time spent in microenvironments (only results with statistical significance *p *< 0.05 were shown)**. The table presents the variation of time spent indoors, outdoors, and in vehicle. Click here for file
