# Supplementary material for: Longitudinal variability of time-location/activity patterns of population at different ages: a longitudinal study in California
Source: Environ Health. 2011 Sep 20;10:80. doi: 10.1186/1476-069X-10-80 (PMC3184256; doi:10.1186/1476-069X-10-80)
Supplement: Additional file 1 — Demographic characteristics of the participants. The table presents the demographic characteristics of the participants in the three age groups. [file 1476-069X-10-80-S1.DOC]

Demographic characteristics of the participants

|  | Children | Parents of young children^a^ | Older adults |
| --- | --- | --- | --- |
| **N** | 150 | 151 | 55 |
| **age** | 1-2 years - 33.3% | 19-24 years - 2.7% | <55 years - 3.6% |
|  | 3-5 years - 57.3% | 25-34 years - 35.1% | 55-64 years - 60.0% |
|  | 6-8 years - 8.7% | 35-44 years - 57.0% | 65-74 years - 18.2% |
|  | 9 years - 0.7% | 45-60 years -5.3% | 75-84 years - 18.2% |
| **Sex** |  |  |  |
| Male | 51.3% | 14.6% | 34.6% |
| Female | 48.7% | 85.4% | 65.5% |
| **Race/Ethnicity** |  |  |  |
| White | 64.0% | 64.2% | 85.5% |
| Asian | 10.7% | 10.6% | — |
| Other | 12.7% | 12.6% | 7.3% |
| Hispanic | 12.7% | 12.6% | 7.3% |
| **Education^b^** |  |  |  |
| High school or lower | | 15.9% | 12.7% |
| College degree or some college | | 51.7% | 61.8% |
| Master, Doctor, and professional degree | | 32.5% | 25.5% |
| **Job status^b^** |  |  |  |
| Employed. |  | 51.0% | 40.0% |
| Stay-at-home parent. | | 37.8% | — |
| Unemployed. |  | 1.3% | 5.5% |
| Retired and have no paid employment. | | 0.7% | 49.1% |
| Other. |  | 7.3% | 5.5% |
| Missing. |  | 2.0% | — |

^a^ One parent did not fill in any information for the child.

^b^ For adults only.
