# Supplementary material for: Longitudinal variability of time-location/activity patterns of population at different ages: a longitudinal study in California
Source: Environ Health. 2011 Sep 20;10:80. doi: 10.1186/1476-069X-10-80 (PMC3184256; doi:10.1186/1476-069X-10-80)
Supplement: Additional file 2 — Variation of time spent in microenvironments (only results with statistical significance p < 0.05 were shown). The table presents the variation of time spent indoors, outdoors, and in vehicle. [file 1476-069X-10-80-S2.DOC]

Variation of time spent in microenvironments (only results with statistical significance *p*<0.05 were shown).

| Variables | Age group | Model^a^ | Day-type^b^ | Season | Longitudinal | Gender^c^ | Age | ICC | O/D correlation |
| --- | --- | --- | --- | --- | --- | --- | --- | --- | --- |
| Indoors^d^ | Overall | O |  | cool>warm |  |  |  | 0.25 | r=0.79  *p*<0.0001 |
|  |  | D | WD>WE | cool>warm | increasing | F>M |  | 0.18 |  |
|  | Children | O |  | cool>warm |  |  |  | 0.23 | r=0.84  *p*=0.0011 |
|  |  | D | WD>WE | cool>warm | increasing |  |  | 0.18 |  |
|  | Parents | O |  | cool>warm |  |  | decreasing | 0.21 | r=0.73  *p*=0.0076 |
|  |  | D | WD>WE | cool>warm |  |  |  | 0.17 |  |
|  | Older | O |  |  |  |  |  | 0.29 | r=0.93  *p*=0.0076 |
|  |  | D |  | cool>warm |  | F>M |  | 0.18 |  |
| Outdoors | Overall | O | WE>WD | warm>cool | decreasing |  | decreasing | 0.26 | r=0.66  *p*<0.0001 |
|  |  | D | WE>WD | warm>cool | decreasing | M>F | decreasing | 0.24 |  |
|  | Children | O |  | warm>cool | decreasing |  |  | 0.29 | r=0.64  *p*=0.0010 |
|  |  | D | WE>WD | warm>cool | decreasing |  |  | 0.22 |  |
|  | Parents | O | WE>WD | warm>cool |  |  |  | 0.26 | r=0.69  *p*=0.0008 |
|  |  | D | WE>WD | warm>cool |  |  |  | 0.21 |  |
|  | Older | O |  | warm>cool |  |  |  | 0.20 | r=0.84  *p*=0.0034 |
|  |  | D |  | warm>cool |  | M>F |  | 0.28 |  |
| In vehicle | Overall | O | WD>WE | warm>cool | increasing |  |  | 0.17 | r=0.43  *p*=0.0035 |
|  |  | D |  |  |  | F>M | increasing | 0.13 |  |
|  | Children | O | WD>WE | warm>cool | increasing |  |  | 0.12 | r=0.50  *p*=0.0587 |
|  |  | D | WE>WD |  |  |  |  | 0.13 |  |
|  | Parents | O | WD>WE |  | increasing |  |  | 0.20 | r=0.49  *p*=0.0275 |
|  |  | D | WD>WE |  |  |  |  | 0.15 |  |
|  | Older | O |  |  | increasing |  |  | 0.22 | r=0.13  *p*=0.6836 |
|  |  | D |  |  |  |  |  | 0.13 |  |
| ^a^ Given that the time-location/activity data having many zero values, mixed-distribution mixed-effects model was used except for time spent on sleep. The mixed-distribution mixed-effects model consists of two parts: whether an individual spent time t a location (occurrence - O) and how long he/she spent at that location (duration – D) (Tooze et al., 2002; Xie et al., 2004). The generalized linear mixed-effects model was used for time spent on sleep.  ^b^ weekday (WD) vs. weekend (WE)  ^c^ Male (M) vs. female (F)  ^d^ Time spent indoors was transformed using (1440 – time spent indoors), thus a zero value means an individual spent a whole day indoors. | | | | | | | | | |
